# Endoplasmic Reticulum Export of GPI-Anchored Proteins

**DOI:** 10.3390/ijms20143506

**Published:** 2019-07-17

**Authors:** Sergio Lopez, Sofia Rodriguez-Gallardo, Susana Sabido-Bozo, Manuel Muñiz

**Affiliations:** 1 Department of Cell Biology, University of Seville, 41012 Seville, Spain; 2 Instituto de Biomedicina de Sevilla (IBiS), Hospital Universitario Virgen del Rocío/CSIC/Universidad de Sevilla, 41012 Seville, Spain

**Keywords:** endoplasmic reticulum, COPII coat, GPI-anchored protein, p24 complex

## Abstract

Protein export from the endoplasmic reticulum (ER) is an essential process in all eukaryotes driven by the cytosolic coat complex COPII, which forms vesicles at ER exit sites for transport of correctly assembled secretory cargo to the Golgi apparatus. The COPII machinery must adapt to the existing wide variety of different types of cargo proteins and to different cellular needs for cargo secretion. The study of the ER export of glycosylphosphatidylinositol (GPI)-anchored proteins (GPI-APs), a special glycolipid-linked class of cell surface proteins, is contributing to address these key issues. Due to their special biophysical properties, GPI-APs use a specialized COPII machinery to be exported from the ER and their processing and maturation has been recently shown to actively regulate COPII function. In this review, we discuss the regulatory mechanisms by which GPI-APs are assembled and selectively exported from the ER.

## 1. Introduction

In eukaryotic cells, the endoplasmic reticulum (ER) is responsible for the synthesis of those luminal and membrane proteins that have to be subsequently distributed by the secretory pathway to their final destination, either outside of the cell or in different secretory and endocytic organelles of the endomembrane system [1]. After insertion through the translocon, newly synthesized secretory proteins undergo conformational folding, assembly, and post-translational modifications including glycosylation and disulfide bond formation. Correctly matured secretory proteins are then selectively packaged as cargos into lipid transport vesicles, which transfer them forward to the next station of the secretory pathway, the ER–Golgi intermediate compartment (ERGIC) in vertebrate cells or the cis-Golgi in other species [2]. These transport vesicles form at specific domains of the ER membrane or ER exit sites (ERES) by its mechanical deformation caused by the polymerization of cytosolic coat protein complex COPII [3,4]. The highly conserved and essential COPII coat complex principally is composed of the two protein heteromeric complexes Sec23/24 and Sec13/31 and the small GTPase Sar1. The sequential assembly of the COPII coat is initiated when Sar1 is activated by its specific Guanosine Exchange Factor, the ER transmembrane protein Sec12. Activated Sar1 recruits first the heterodimer Sec23/24 to the ERES. Then, the subcomplex Sar1-GTP-Sec23/24 selectively binds the secretory cargo (via the cargo-adaptor COPII subunit Sec24) forming the inner COPII coat layer that, in turn, recruits the heterotetramer Sec13/31 or outer COPII coat layer. This outer layer polymerizes to form a cage-like structure that finally sculpts the ER membrane into a COPII vesicle at discrete spatial regions on the ER membrane termed ER exit sites (ERES) [5,6]. The structural and functional organization of the ERES is, however, still poorly defined but depends on the presence of Sec16, a large multidomain peripheral protein. Sec16 has been proposed to oligomerize in a preformed cluster that would act as a scaffold for COPII vesicle budding by binding COPII coat components. In addition, Sec16 is also thought to act together with Sec12, the activator of the GTPase Sar1, to regulate the activity of Sar1 and so they can modulate the assembly of the COPII coat by recruiting first the inner layer Sec23/Sec24 and subsequently the external layer Sec13/Sec31 [7,8,9]. 

Although the basic mechanism of COPII function is relatively well known, several key questions remain to be answered. For example, the fact that a third of all eukaryotic proteins enter the ER to be exported towards the secretory/endocytic endomembrane system raises the question of how the COPII system can accommodate a large variety of cargoes in size, structure, and topology. Another key question is how the COPII system is regulated to be adapted to different needs for cargo secretion, which occurs in many specialized secretory cells. The study of the ER export of some specific secretory cargoes such as the glycosylphosphatidylinositol (GPI)-anchored proteins (GPI-APs) is contributing to address these important questions [10]. GPI-APs constitute a category of lipid-linked secretory proteins with special biophysical properties that leads them to be differentially trafficked from other secretory proteins. They require specialized COPII machinery for exit from the ER. Furthermore, the maturation of these cargo proteins has been recently shown to actively regulate the COPII function [11]. In this review, we discuss the regulatory mechanisms by which GPI-APs are assembled and exported from the ER by a specialized COPII machinery in yeast and mammalian cell systems.

## 2. Brief Overview of GPI-AP Biosynthetic Pathway

GPI-APs are expressed from yeast to human and consist of a luminal secretory protein linked to a glycolipid or GPI anchor through which it is attached to the external leaflet of the plasma membrane [12]. The GPI-anchor precursor is formed by a phospholipid moiety with a glycan backbone, which is made by a complex series of sequential reactions at the ER membrane. Newly synthesized proteins containing a GPI attachment signal sequence at their C terminus receive then the GPI anchor in the lumen of the ER as a conserved posttranslational modification [13]. Once attached to the protein, the GPI anchor undergoes a structural modification or remodeling by which is transformed into an active trafficking signal that, in a regulated manner, exports GPI-APs from the ER to the plasma membrane to play vital physiological roles [11,14]. In mammalian cells, the GPI-AP family is represented by more than 150 different proteins including enzymes, adhesion molecules, receptors, protease inhibitors, transcytotic transporters, and complement regulators. Because of this wide range of key biological activities performed by GPI-APs, they have been found to be essential in processes such as embryogenesis, development, neurogenesis, fertilization, and the immune system, being involved in the development of prominent human diseases, including neurodegeneration and cancer, underscoring their clinical relevance [15,16]. The yeast *Saccharomyces cerevisiae* has more than 60 GPI-APs, which are essential for growth. A subset of yeast GPI-APs remain attached to the plasma membrane, but many others undergo shedding, being released and subsequently incorporated into the cell wall [17,18].

## 3. GPI Anchor Synthesis and Attachment in Yeast and Mammalian Cells

GPI anchor attachment is a unique post-translational modification of proteins by glycolipid in eukaryotes (Table 1). The highly conserved core structure of the GPI anchor precursor comprises a phospholipid moiety acyl-phosphatidylinositol (acyl-PI) and a glycan backbone made of a glucosamine (GlcN) and three or four mannoses (Man), in which Man1, Man2, and Man3 have an ethanolamine phosphate (EtNP) side-branch. The GPI anchor synthesis involves more than 20 genes and begins at the cytosolic side of the ER membrane when an UDP-N-acetyl-GlcN (UDP-N-GlcNAc) is transferred to a phosphatidylinositol (PI) by the protein complex GPI-GlcNAc transferase, of which the mammalian/yeast members are PIG-H/Gpi15, PIG-Y/Eri1, PIG-A/Gpi3, PIG-P/Gpi19, PIG-C/Gpi2, and PIG-Q/Gpi1 [13]. Then, the acetyl group of GlcNAc is enzymatically removed by the deacetylase PIG-L/Gpi12 up to GlcN-PI that it is translocated to the luminal side of the ER membrane. Such translocation is thought to be mediated by a flippase activity that remains to be identified. Inside the ER, the acyltransferase PIG-W/Gwt1 adds a palmitoyl group to the position 2 of the inositol ring generating GlcN-(acyl)PI [13]. In mammalian cells, GlcN-(acyl)PI undergoes a specific lipid remodelling process that converts the diacyl-PI moiety to a mixture of 1-alkyl-2-acyl PI, major form, and diacyl-PI, minor form [12]. In yeast, only diacyl-PI has been described. Afterwards, two mannosyl transferases, named GPI mannosyl-transferase 1 (PIG-M/Gpi14 and PIG-X) and GPI mannosyl transferase 2 (PIG-V/Gpi18), add two Man to the GPI using dolichol-P-Man as substrate. Next, EtNP transferase enzyme 1 (PIG-N/Mcd4) adds the first EtNP of the GPI on Man1 [17] and GPI mannosyl transferase 3 (PIG-B/Gpi10) adds the Man3. The next step is the addition of Man4 by PIG-Z/Smp3 before the addition of the bridging EtNP to Man3, that links the GPI to proteins, by EtNP transferase enzyme 2, consisting of the catalytic subunit PIG-O/Gpi13 and the stabilizing subunit PIG-F/Gpi11 [13]. Finally, an EtNP to Man2 is added by the EtNP transferase enzyme 3, consisting of the catalytic subunit PIG-G/Gpi7 and the stabilizing subunit PIG-F/Gpi11 [17,19,20].

Once the GPI anchor has been fully synthesized, the C-terminal of the protein is attached to the amine group of the bridging EtNP in a reaction of transamidation by the GPI transamidase complex that contains five essential proteins (the catalytic core: PIG-K/Gpi8 and another four subunits, PIG-S/Gpi17, PIG-T/Gpi16, PIG-U/Gab1 and GPAA1/Gaa1). The transamidation reaction requires a previous cleavage of the protein by the caspase-like activity of PIG-K/Gpi8p [12]. GPI attachment is essential for efficient ER export of GPI-APs. Unanchored precursor proteins are not actively incorporated into the COPII vesicles because the ER export machinery cannot recognize them [21]. However, due to the intensive vesicular export flux, the unanchored precursor proteins can passively leave the ER by a default pathway or bulk flow and arrive to the Golgi, from where they can be retrieved back to the ER in COPI coated vesicles. This retrieval is driven by the cargo receptor Rer1 that recognizes the short transmembrane domain of unanchored precursor proteins and links them with the COPI coat for their effective recycling to the ER [22].

## 4. GPI-APs Use a Specialized Mechanism for Selective ER Export

A key question in membrane trafficking is how the COPII system can accommodate a large diversity of secretory protein cargoes. GPI-APs represent an exceptional model to better understand how lipid-associated luminal cargo proteins, including the lipid-associated morphogens Hedgehog, Wnt, or Ephrin [23], can be efficiently exported from the ER. After GPI attachment to the nascent protein, GPI-APs must be exported from the ER in lipid vesicles generated by the polymerization of the COPII coat at the ERES. However, the luminal and lipidic nature of GPI-APs challenges the efficient operativity of the COPII system. Indeed, GPI-linked cargo crowding in the luminal side of the ER has been shown to oppose the ER membrane bending achieved by the COPII coat for vesicle budding. Furthermore, the presence of the GPI-lipid appears to increase the ER membrane rigidity which complicates its bending. This is particularly relevant for yeast cells since they have to export GPI-APs with very long saturated acyl chains from the ER. These special GPI-lipids form rigid lipid domains that lead yeast GPI-APs to be segregated and sorted from other secretory proteins into specific ERES and COPII vesicles. Finally, the cytosolic COPII system cannot directly capture GPI-APs since they are entirely luminal cargo proteins. To cope with all these inherent difficulties, GPI-APs need to use a special mechanism of ER export that involves specialized machinery such as a transmembrane COPII coat adaptor or cargo receptor and specific isoforms of the conventional Sec24 COPII cargo-binding subunit (Table 2). The access of GPI-APs to the ERES and their recognition by its specialized ER export machinery is regulated by the structural modification or remodeling of the lipid and glycan parts of the GPI anchor (Table 2). Below we discuss the specialized mechanisms by which GPI-APs are selectively exported from the ER in yeast and mammalian systems.

### 4.1. Lipid-Based Sorting of GPI-APs into Specific ERES

**Figure 1:**
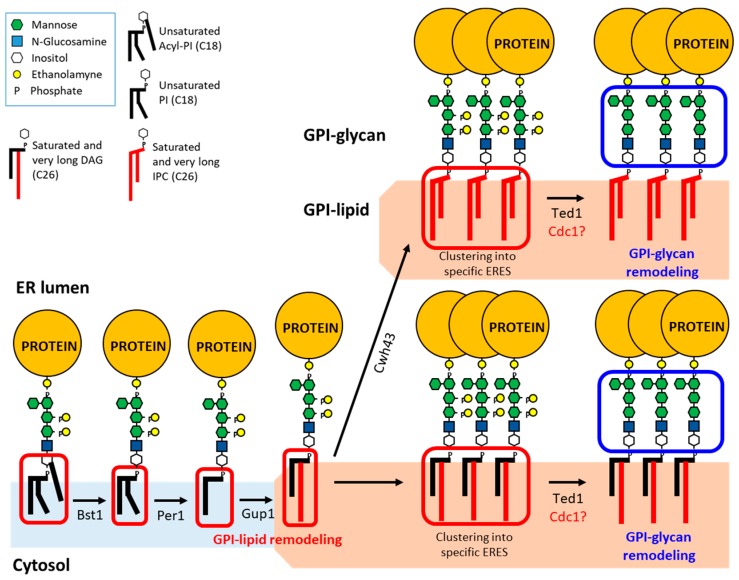
GPI anchor remodeling in yeast. The GPI anchor is synthesized in the ER and transferred to proteins by the GPI–transamidase complex. After protein attachment, the GPI-lipid undergoes a structural remodeling that in yeast occurs almost entirely at the ER. First, the inositol is deacylated by Bst1. Next, the fatty acid is remodeled by Per1 and Gup1, which remove the unsaturated fatty acid at the sn2 position and replace it with a very long-chain and highly saturated fatty acid (C26:0), respectively. In most cases, the C26:0 diacylglycerol (DAG) generated is swapped with a very long-chain saturated (C26:0) ceramide yielding (C26:0) inositolphosphoceramide (IPC). In both C26:0 DAG and C26:0 IPC-based GPI-APs, the side-chain EtNP on Man2 is removed by Ted1. This reaction is critical for subsequent recruitment of the ER export machinery. The side-chain EtNP on Man1 is also removed from some fractions of GPI anchors by Cdc1 [14] but it remains unclear which GPI-APs undergo this process. After GPI-APs are transported to the Golgi, additional Man is transferred to the Man4 (not shown). Once arrived at the cell surface, GPI-APs with C26:0 DAG as GPI-lipid are cleaved and cross-linked to α1,6-glucans on the cell wall, whereas GPI-APs with C26:0 IPC remain at the plasma membrane (not shown).

Once the GPI anchor has been attached to the protein, the lipid and glycan parts of the GPI anchor undergo a structural remodeling. Specifically in yeast, the GPI-lipid remodeling occurs entirely in the ER (Figure 1) [17], leading to the replacement of the initial short and unsaturated acyl chain lipid precursor moiety (C18:1 acyl-PI) with a very long and highly saturated acyl chain lipid moiety (C26:0 diacylglycerol (DAG) or C26:0 inositolphosphoceramide (IPC)). This complex biochemical process is initiated with the inositol deacylation by the remodeling enzyme Bst1 [25]. Next, the fatty acid is remodeled which comprises the removal of the short and unsaturated fatty acid (C18:1) at the sn2 position by Per1 [26] and its replacement with a very long-chain saturated fatty acid (C26:0) by Gup1 [27]. The C26:0 DAG generated as the GPI-lipid is thought to be present only in those GPI-APs destined to be transferred to the cell wall after their shedding from the plasma membrane [28]. However, the GPI-APs, whose fate is to remain in the plasma membrane, replace the C26:0 DAG with ceramide that also contains a very long-chain saturated fatty acid (C26:0) by Cwh43 generating IPC (Figure 1) [28,29]. Therefore, the type of remodeled lipid moiety of GPI-APs (C26:0 DAG or C26:0 IPC) contributes to determine their final destination in yeast, although the molecular basis for this is unknown [28].

**Figure 2:**
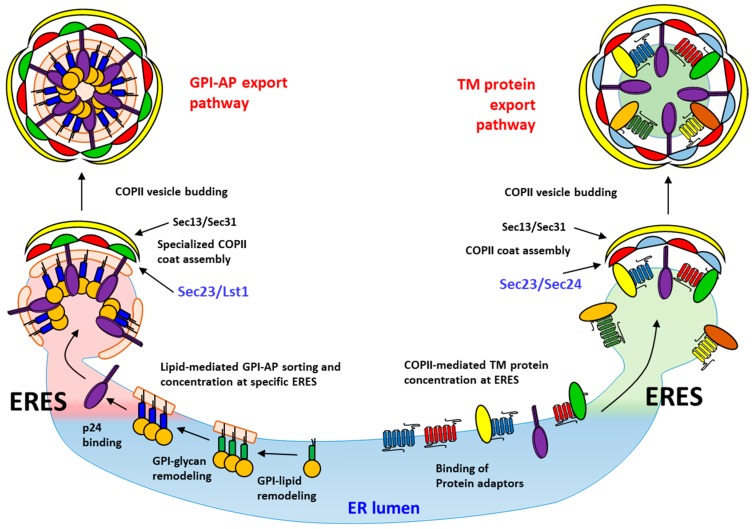
GPI-AP sorting upon ER exit in yeast. A schematic model for the sorting mechanism of GPI-APs at the ER in yeast cells is shown. Upon GPI-lipid remodeling with very long and saturated fatty acid chains in the ER, GPI-APs are segregated from transmembrane secretory proteins and concentrated into specific ERES. Next, the GPI-glycan remodeling allows the subsequent recruitment of the p24 complex, which functions as a specific lectin by recognizing the remodeled GPI-glycan moiety of GPI-APs, to these ERES. This binding stimulates p24 receptor to recruit a specialized COPII coat that leads to the formation of COPII vesicles enriched in GPI-APs. By contrast, transmembrane secretory cargo proteins are concentrated into distinct ERES than GPI-APs by a COPII-dependent mechanism that involves their capture by the COPII coat directly or indirectly through cargo receptors. The p24 complex has been also shown to follow the non-GPI-AP pathway to the Golgi. Part of images from Motifolio drawing toolkits (http://www.motifolio.com/) were utilized in the figure preparation.

The presence of a very long and saturated acyl chain lipid into the GPI anchor such as C26:0 DAG or C26:0 IPC has been proposed to endow GPI-APs with a particular way of binding to the ER membrane that leads them to be separated from other secretory membrane proteins upon export from the ER [30]. Although for long time it was thought that the first protein sorting step in the secretory pathway takes place at the Golgi, from where secretory cargo are segregated and delivered to their functional destinations, pioneering studies showed that, in yeast, GPI-APs are segregated from other cargoes at the level of the ER and sorted into specific ERES and COPII vesicles (Figure 2) [31,32]. This conclusion was reached after using two independent isolation methods to separate different COPII vesicles generated from an in vitro vesicle budding assay [32]. A following study confirmed that sorting of GPI-APs occurred upon the ER exit in vivo by using a genetic sorting assay which employs thermosensitive COPII mutant yeast cells that accumulate secretory cargo in the ERES at restrictive temperature [31]. Specifically, it was observed that GPI-APs accumulate in ERES that are distinct from those in which other secretory proteins accumulate. This ER sorting of GPI-APs in yeast could be driven by a lipid-based sorting mechanism based on the acquisition of a very long and saturated acyl chain lipid as a consequence of the GPI-lipid remodeling [10,30,33]. Indeed, lipid remodeling mutants, such as *bst1∆*, *per1∆*, or *gup1∆*, accumulate unremodeled GPI-APs with an unsaturated and short acyl chain lipid (C18:0) that cannot enter the ERES [31]. Furthermore, only remodeled GPI-APs can be biochemically detected in detergent-resistant membrane (DRM) fractions, which has been suggested to reflect their aggregation into lipid domains enriched in ceramides [21,25,26]. Indeed, ceramide synthesis is specifically necessary for GPI-AP association with the ER membrane and for ER export [34,35]. Moreover, biophysical experiments performed in liposomes suggest that ceramides can form clusters with special physical characteristics [36]. Moreover, in yeast, ceramide is mainly exported from the ER in COPII vesicles and sphingolipid synthesis at the Golgi depends on the biosynthesis of the GPI anchor [37,38]. Taken together, these results suggest that lipid-remodeled GPI-APs and ceramides could be co-sorted together into the same specialized ERES and COPII vesicles. The presence of a very long saturated fatty acid (C26:0) in GPI-APs and ceramides in contrast to the glycerolipids (C16:1 and C18:1) present in the ER could promote their selective concentration by self-assembly, creating specific microdomains at the ER membrane. These domains of very long and saturated lipids might produce an unfavorable environment for transmembrane proteins in the ER membrane, which could lead to the formation of specialized ERES by exclusion. Lipid-remodeled GPI-APs would be then transported from these ERES to the Golgi in specialized COPII vesicles (Figure 2).

In mammalian cells, unlike yeast, GPI-APs do not appear to be sorted during the export from the ER. Indeed, they have been found using conventional microscopy to be concentrated in the same ERES together with other secretory proteins, at least with the resolution available at the time of this study [39]. This could be due to the fact that, although in mammalian cells GPI-lipid remodeling also begins in the ER after GPI-deacylation by the Bst1p orthologue PGAP1 (Figure 4) [25], the subsequent fatty acid remodeling by which the GPI anchor acquires a saturated lipid occurs later at the Golgi. There, the Per1 orthologue PGAP3 removes the unsaturated fatty acid from the sn-2 position to form lyso-GPI [40]. Next, although a mammalian orthologue of yeast Gup1 has not been identified, the acyl-transferase activity of PGAP2 adds a C18:0 fatty acid into the sn-2 position of the GPI-lipid [41]. Interestingly, this remodeling, by which the GPI-lipid is converted in a saturated lipid, spatially correlates with the sorting process that GPI-APs undergo at the Golgi [30,40]. Indeed, mammalian GPI-APs are segregated and sorted from other secretory cargoes at the level of the Trans-Golgi Network (TGN) from where they are separately routed to the cell surface [42,43,44]. In particular, in most polarized epithelial cells, GPI-APs are sorted at the TGN to the apical plasma membrane. This sorting process is driven by GPI-AP oligomerization, which in turn has been shown to occur in protein–protein and cholesterol-dependent manners [45,46,47,48]. In addition to the protein ectodomain of GPI-APs and the surrounding lipid environment, the GPI anchor might also play a relevant role in the oligomerization and sorting of GPI-APs in polarized epithelial cells. The acquisition of long and saturated fatty acids by GPI lipid remodeling at the Golgi could initially lead to the lateral association of GPI-APs into saturated lipid domains at the TGN that are further stabilized by oligomerization through protein–protein interactions between the protein ectodomains of GPI-APs. Oligomerized GPI-APs would be then concentrated in lipid platforms that lead them to be segregated from other secretory proteins and sorted into specific TGN export sites for apical surface delivery. Interestingly, this TGN sorting process has been shown to regulate both GPI-AP organization and function at the apical membrane [45]. Furthermore, it has been shown that remodeled GPI-lipids with long saturated acyl chains are required to form nanoclusters of GPI-APs at the plasma membrane of mammalian cells [49,50]. Taken together, these data suggest a relevant role for GPI-lipid remodeling in GPI-AP sorting at the TGN. Therefore, the lack of GPI-lipid remodeling at the ER would explain why GPI-APs are not sorted upon ER exit in mammalian cells [30]. For the same reason, the fact that GPI-lipid remodeling occurs at the ER in yeast suggest that both GPI-AP sorting steps, at the ER in yeast or at the TGN in mammalian cells, could be driven by similar GPI-lipid remodeling-based mechanisms. Nevertheless, to address this possibility, it would be required to analyze whether, in yeast, protein–protein interactions also promote clustering and oligomerization of lipid-remodeled GPI-APs at the ER.

### 4.2. A Specialized COPII System Drives GPI-AP Export from the ER

In yeast, once the lipid remodeling of the GPI-anchor has finished and GPI-APs have been subsequently sorted and concentrated at the specific ERES, they must interact with the cytosolic COPII coat in order to be efficiently incorporated into COPII vesicles for Golgi delivery. However, because GPI-APs are completely luminal proteins and do not span the ER membrane, packaging into nascent COPII vesicles requires the help of a transmembrane cargo–coat adaptor or cargo receptor. This role of selective COPII coat adaptor for GPI-APs is accomplished by proteins of the conserved p24 family [21,33,51,52]. The p24 proteins are transmembrane proteins with type I topology. They have a short cytosolic tail with a strong COPII binding signal [53,54]. Several distinct p24 proteins assemble through their luminal coiled-coil domains in a heteromeric complex that continuously cycles between ER and Golgi compartments [55]. In yeast, the p24 complex consists of at least four members of the p24 family; Emp24, Erv25, Erp1, and Erp2 [56]. Several studies have shown a direct role of the yeast p24 complex as cargo receptor in the ER exit of GPI-APs [11,21,33]. First, the efficient ER-to-Golgi transport of GPI-APs depends on the p24 complex. Second, the in vitro ER budding of GPI-APs directly and selectively requires the p24 complex. Third, GPI-APs can be directly cross-linked to the p24 proteins in purified COPII vesicles. Finally, GPI-APs are indirectly connected through the p24 complex with a Lst1, a paralog of the COPII Sec24 cargo-binding subunit [9]. Interestingly, Lst1 is specifically required for the selective ER export of GPI-APs and contains specific binding sites for the p24 proteins. Moreover, disruption of these specific sites on Lst1 resulted in the accumulation of GPI-APs at the ER. Therefore, taken together, the experimental data indicate that the p24 complex works as an adaptor that connect GPI-APs to a specialized COPII coat. Nevertheless, the fact that GPI-AP cargo clustering does not require the p24 complex and GPI-APs are recognized by the p24 complex after their GPI-lipid remodeling suggests that the p24 complex acts as an adaptor after GPI-lipid remodeling-based sorting at ERES [21]. Therefore, the p24 complex must be then attracted to GPI-AP containing ERES to link them with a specialized COPII coat for efficient COPII vesicle packaging (Figure 2).

The biogenesis of specialized COPII vesicles from specific GPI-AP containing ERES in yeast involves the recruitment of a specialized ER export machinery, including the p24 complex and the COPII coat subunit Lst1. The need to use this specialized COPII machinery appears to be due to the luminal topology of both the p24 complex and GPI-APs which, when locally concentrated at specific ERES, impose special biophysical requirements for COPII-coated vesicle budding [57,58]. In particular, it is especially relevant that the size of the GPI anchors is short in relation to the heavily glycosylated luminal ectodomains of GPI-APs. This difference generates a negative curvature force associated with membranes enriched in GPI-AP that appears to be overcome by the external COPII coat subunit Sec13 by providing enough rigidity to the COPII coat [59]. In addition, Sec13-dependent COPII coat rigidity has been proposed to be adjusted by Lst1, which creates a COPII vesicle bud with a larger diameter. Consistently, it has been shown that COPII vesicles produced in vitro with mixed Sec24-Lst1 coats are slightly larger than those vesicles formed only by Sec24. Thus, Lst1 and Sec13 could specifically cooperate to capture larger cargos, such as clusters of lipid remodeled GPI-APs inserted into rigid ceramide-enriched membranes by providing the COPII coat with a specialized scaffold [9,60]. This idea is supported by the fact that p24 and GPI-AP remodeling mutants bypass the essential requirement for Sec13 [61]. Therefore, the p24 complex specifically binds remodeled GPI-APs to recruit and stabilize Lst1 at specific ERES and thus enable the formation of the COPII vesicles for effective GPI-AP export from the ER.

In mammals, like in yeast, GPI-APs are exported from the ER by a specialized machinery, that also involves the p24 complex [51,52]. GPI-APs are selectively recognized by the mammalian p24 complex in order to be connected to the COPII coat for efficient ER export in COPII vesicles (Figure 4). Interestingly, the membrane-adjacent α-helical binding domain of the p24 proteins has been involved as the binding domain of the mammalian p24 proteins [62]. However, unlike yeast, incorporation and concentration of mammalian GPI-APs into ERES have been shown to depend on the p24 complex [52]. This p24 complex dependence suggests that GPI-AP concentration at ERES is not mediated by a lipid-based sorting mechanism but instead by a direct COPII-dependent capture mechanism, since the p24 connects the GPI-AP in the ER lumen with the COPII in the cytosol and ERES are domains for COPII assembly. Thus, GPI-APs are captured by the p24 complex and linked with the COPII coat during its assembly, which leads to their concentration at ERES. Despite these differences in mechanisms of GPI-AP concentration at ERES between the mammalian and yeast cells, imposed by the GPI anchor lipid composition, mammalian GPI-APs also use, like in yeast, a specialized COPII coat. Indeed, Sec24C and Sec24D, the specific isoforms of the COPII cargo binding subunit Sec24, are required for the ER export of both GPI-APs and the p24 complex [51] suggesting that, similarly to yeast, the mammalian p24 complex interacts with the inner layer of the COPII coat through these specific COPII subunits for efficient packaging of GPI-APs into COPII vesicles.

## 5. COPII Function is Actively Regulated by GPI-Glycan Remodeling

**Figure 3:**
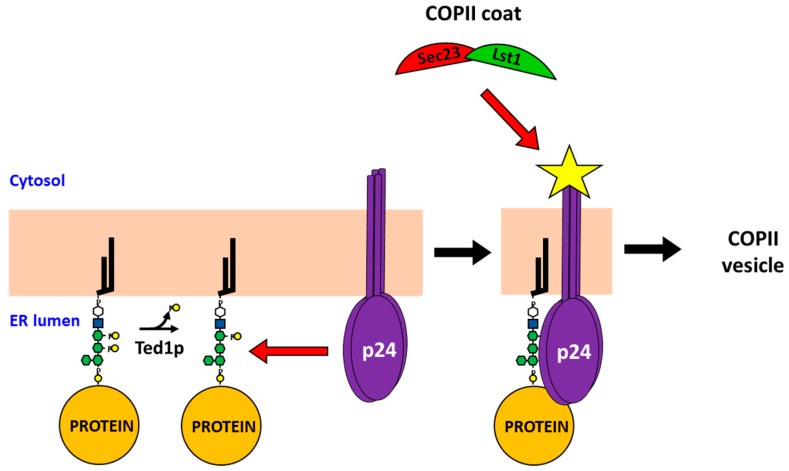
GPI-anchor remodeling actively regulates the recruitment of a specialized COPII coat in yeast. The removal of the EtNP on the second mannose of the GPI-glycan by Ted1 triggers the interaction of the GPI-AP cargo with the p24 receptor. This binding modifies the conformation of the p24 receptor (represented by the yellow star) which prompts the selective recruitment of the of the specific inner COPII coat Lst1/Sec23 and subsequent COPII vesicle budding. This mechanism implies that formation of these specialized COPII vesicles is fine-tuned by the amount of GPI-APs that are ready to be exported from the ER.

The study of GPI-AP trafficking is also contributing to answering the relevant question in membrane trafficking of how the COPII system can adapt to different cellular secretory needs. In this sense, it has been reported that, in yeast, a specialized COPII system actively responds to the presence of mature GPI-APs which are ready to be exported from the ER. This system requires the structural remodeling of the glycan part of the GPI-anchor [11]. GPI-glycan remodeling is a conserved process by which the initial side-chain EtNP present on the second mannose of the GPI-glycan is removed by the specific phosphodiesterase Ted1 in yeast. Interestingly, the GPI-glycan remodelase activity of Ted1 is required for the selective GPI-AP recognition by the p24 complex receptor in vivo. Moreover, the p24 complex has been shown to bind, in vitro, a synthetic glycan composed of Man4-Man3-Man2-Man1-GlcN-InoP without the EtNP on the second mannose that mimics the remodeled GPI-glycan. Importantly, the fact that mannose can outcompete this specific interaction indicates that the p24 complex functions as a specific lectin by recognizing the remodeled structure of the GPI-glycan. Most importantly, this lectin-based recognition of the remodeled GPI-glycan by the yeast p24 complex has been shown to trigger the p24 complex recruitment and stabilization of Lst1 to ERES (Figure 3) [11]. Thus, the GPI-glycan remodeling actively regulates the p24 complex to connect mature GPI-APs specifically with Lst1 but not with Sec24p, despite the fact that both subunits can be bound in vivo by the p24 proteins. Since the p24 complex has been shown to exit the ER for Golgi delivery in the non-GPI-AP-containing COPII vesicles, in addition to the specialized GPI-AP-containing COPII vesicles, it is conceivable that the p24 complex binds Lst1 and Sec24 in ERESs for GPI-APs and non-GPI-APs, respectively. Although the underlying mechanism for this dual specificity of the p24 complex towards Lst1 and Sec24 in yeast is still unknown, it is plausible that the involvement of a conformational change of the p24 proteins upon direct binding to the GPI-glycan increases their affinity for Lst1. This potential conformational change could be also greatly influenced by the lipidic environment of GPI-AP containing ERES, most likely enriched with highly saturated and very long acyl chain lipids such as C26:0 ceramides.

Most importantly, the observation that Lst1 recruitment is specifically triggered by GPI-glycan remodeling indicates that the recruitment of the COPII coat by cargo receptors is not constitutive but instead is actively regulated by binding of mature ligands. This implies that COPII function can be controlled by luminal cargo maturation, supporting the notion that secretory cargo plays an active role in vesicular transport instead of simply being a passive traveler. It also reveals a novel functional link between luminal cargo maturation and COPII vesicle budding, providing a mechanism to adjust specialized COPII vesicle production to the amount and quality of their luminal cargos that are ready for ER exit. This could help to better understand how the COPII system can be regulated to meet different needs for secretion of luminal cargo.

**Figure 4:**
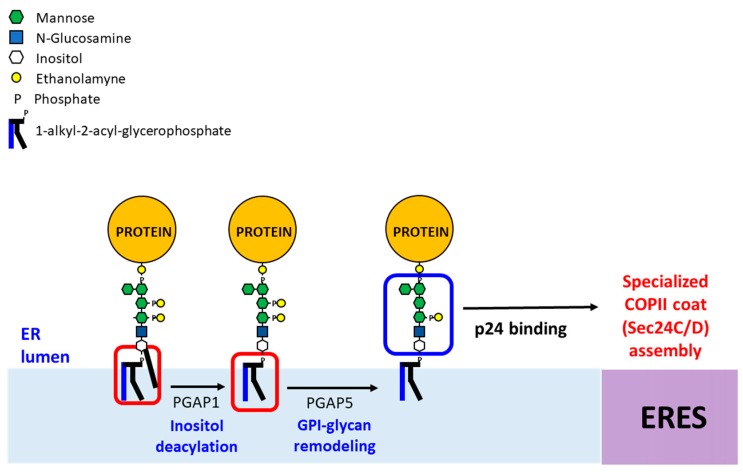
GPI anchor remodeling in mammalian cells at the ER. Newly synthesized GPI-APs undergo two remodeling reactions, inositol-deacylation by PGAP1 and removal of the EtNP side chain from Man2 by PGAP5 in the ER. Next, the p24 complex recognizes the remodeled GPI-APs and connect them with the COPII coat for ER export in COPII vesicles. In the Golgi, GPI-APs undergo fatty acid remodeling by PGAP3 and PGAP2, generating mature GPI-APs (not shown).

In mammalian cells, the GPI-glycan is structurally remodeled immediately after GPI anchor deacylation by PGAP1. Like in yeast, GPI-glycan remodeling involves the removal of the EtNP on the second mannose of the GPI-glycan by the Ted1 orthologue PGAP5 and regulates the specific binding and recognition of remodeled GPI-AP by the p24 complex (Figure 4) [63]. Therefore, due to its high degree of conservation, the remodeling of the GPI-glycan could also actively regulate the COPII function in mammalian cells, although this remains to be addressed experimentally. Nevertheless, it is tempting to propose that secretory cells could use an analogous mechanism to control the production of luminal lipid-modified secretory cargoes such as Hedgehog, Ephrin, or Wnt, depending on their demands. In agreement with this possibility, the p24 proteins have been shown to be required for secretion of Wnt ligands [64,65,66,67].

## 6. Quality Control and ER Export of Misfolded GPI-APs

Once inserted in the ER through the translocon, newly synthesized secretory proteins undergo native conformational folding with the aid of chaperones before they are exported from the ER. The correct folding state of secretory proteins is monitored by a sophisticated ER quality control that can detect the presence of terminally misfolded proteins and send them for degradation to preserve the functional homeostasis of the secretory pathway. Soluble and transmembrane misfolded proteins are retained in the ER and eliminated by a conserved process, called ER-associated degradation (ERAD). ERAD substrates are retro-translocated into the cytosol and polyubiquitinated by the multispanning ubiquitin ligase Hrd1 and subsequently degraded by the proteasome [68]. However, studies in yeast have shown that misfolded GPI-APs are not target to the ERAD pathway for proteasomal degradation. Instead, misfolded GPI-APs, once remodeled, are rapidly recognized by the p24 complex to prevent their capture by Hrd1 and exported from the ER to be ultimately delivered to the vacuole for degradation [69,70]. Studies in mammalian cells using the misfolded form of the prion protein (PrP*) have arrived at the same conclusion as PrP* it is not degraded by ERAD and exit the ER despite their misfolding [71]. Furthermore, PrP* requires the p24 complex for ER export and travels via the plasma membrane to the lysosomes for degradation [72]. Indeed, it was observed that although PrP* is primarily retained in the ER at steady state, it becomes rapidly released into the secretory pathway by acute ER stress in a process called RESET (rapid ER stress-induced export), that relaxes the ER stress until the unfolded protein response (UPR) is fully active [72]. Interestingly, a recent study has shown that PrP* exits the ER and travels via the cell surface to lysosomes in a complex with resident ER chaperones and p24 proteins [73]. One of the ER chaperones that escort PrP* is calnexin, which has been recently shown to play a dual role in the processing and maturation of GPI-APs. Calnexin binds with N-glycans on GPI-APs to promote protein folding and retains GPI-APs in the ER to assist efficient inositol-deacylation of GPI-APs by PGAP1 for correct processing of the GPI anchor moieties [74].

## 7. Disorders Associated with the ER Export of GPI-APs

Over 150 mammalian GPI-APs are expressed at the cell surface where they play diverse physiological roles in cell signaling, cell adhesion and migration, cell metabolism, embryonic development, or immune response [12]. Therefore, it is not surprising that mutations preventing the efficient ER export of GPI-APs cause congenital disorders. These mutations have been found in PIG (phosphatidyl inositol glycan) genes encoding enzymes involved in the biosynthesis and attachment of the GPI anchor, and PGAP (post GPI attachment to proteins) genes encoding GPI-anchor remodeling enzymes [12], most of them required for the efficient ER export of GPI-APs. To date, it has been reported mutations in at least 13 PIG and PGAP genes that cause GPI-associated congenital disorders [16]. All these disorders are autosomal recessive, except the PIG-A-associated disease which is X-linked recessive. Clinical phenotypes are often variable, although they share overlapping features including developmental delay, seizures, hypotonia, weakness, ataxia, and dysmorphic features. Interestingly, in some instances, different mutations within the same gene can produce multiple phenotypes. For example, mutations in PIG-A can cause at least five phenotypically distinct disorders: paroxysmal nocturnal haemoglobinuria, a rare hematological disorder in which red blood cells break down earlier than normal due to defective self-protection against complement [75], X-linked syndrome associated with neurodegeneration, cutaneous abnormalities, and systemic iron overload, multiple congenital anomalies-hypotonia-seizures syndrome 2, a severe syndromic form of X-linked intellectual disability, and early-onset epileptic encephalopathies. The reason why different mutations within the same gene produce distinct types of disorders is unknown, although this could depend on how severe the mutation is or how the mutation influences the activity of other GPI biosynthetic enzymes [76].

## 8. Conclusions

The special mode of association of GPI-APs with the ER membrane leads them to be differently exported from the ER. In yeast, GPI-APs exit the ER via a specific COPII-dependent pathway. They undergo a previous sorting event that could be imposed by the acquisition of a very long and highly saturated lipid during the remodeling of the GPI anchor. The special biophysical properties of this lipid could lead to the clustering and concentration of GPI-APs into specific ERES and COPII vesicles. Consistently, mammalian GPI-APs are sorted later in the Golgi where the GPI anchor incorporates the saturated fatty acid upon lipid remodeling. Nevertheless, the use of high-resolution microscopy techniques should clarify whether mammalian GPI-APs are also sorted at the level of the ER due to their specialized ether–lipid-based structure. Another important issue is that GPI anchoring confers a complete luminal topology to GPI-APs which hinders their exit from the ER, making the use of a specialized COPII coat involving Lst1 in yeast and Sec24C/D in mammals necessary. Also critical for GPI-AP trafficking are GPI-anchor synthesis and remodeling processes. It is possible that the subcellular distribution of enzymes that catalyze these two sophisticated processes plays a key role in the differential ER export of GPI-APs. Indeed, recent data show that PIG-B, a GPI anchor synthesis enzyme, is specifically localized in the nuclear envelope of Drosophila cells [77]. Finally, it is important to highlight that, in yeast, the GPI-glycan remodeling by Ted1 triggers the recruitment of Lst1, indicating that cargo maturation actively regulates the generation of specialized COPII vesicles which efficiently export mature GPI-APs from the ER. The fact that remodeling of the GPI-glycan occurs after protein anchoring indicates that the formation of specialized COPII vesicles is adjusted to the amount of proteins that are properly attached to the GPI anchor and ready for ER export. It will be important to determine whether this active regulation of the COPII coat by cargo maturation observed in yeast is a general phenomenon. The existence of this mechanism could provide insight into how the ER export can be regulated to meet changing demands for transport of lipid-associated luminal cargoes, including GPI-APs and lipid-associated morphogens.

## Figures and Tables

**Table 1:** Enzymes involved in the synthesis and attachment of the GPI-anchor in yeast and mammalian cells.

Process	Enzymes	
	Yeast	Mammals
Synthesis of the GPI Anchor		
Transfer of GlcNAc to PI	GPI-GlcNAc transferase complex: Gpi1, Gpi2, Gpi3, Gpi15, Gpi19, Eri1	GPI-GlcNAc transferase complex: PIG-Q, PIG-C, PIG-A, PIG-H, PIG-P, PIG-Y
Deacetylation of GlcNAc-PI	Gpi12	PIG-L
Inositol acylation of GlcN-PI	Gwt1	PIG-W
Addition of Man1 to GlcN-PI	Gpi14	PIG-M and PIG-X
Addition of Man2 to Man1	Gpi18	PIG-V
Addition of EtNP to Man1	Mcd4	PIG-N
Addition of Man3 to Man2	Gpi10	PIG-B
Addition of Man4 to Man3	Smp3	PIG-Z
Addition of the bridging EtNP to Man3	Gpi13, Gpi11	PIG-O, PIG-F
Addition of EtNP to Man2	Gpi7, Gpi11	PIG-G, PIG-F
**GPI anchor attachment to the protein**	GPI transamidase complex: Gpi8, Gpi17, Gpi16, Gab1, Gaa1	GPI transamidase complex: PIG-K, PIG-S, PIG-T, PIG-U, GPAA1

**Table 2:** Components of the specialized remodeling and ER export machinery of GPI-APs in yeast and mammals.

Process	Enzymes	
	Yeast	Mammals
**Lipid remodeling of the GPI anchor**		
Inositol deacylation	Bst1	PGAP1
sn2 deacylation	Per1	PGAP3
sn2 reacylation with long saturated fatty acid	Gup1	PGAP2
Replacement of C26:0 diacylglycerol with C26:0 ceramide	Cwh43	
**Glycan remodeling of the GPI anchor**		
Removal of EtNP from Man2	Ted1	PGAP5
Removal of EtNP from Man1	Cdc1	
**COPII coat recruitment**		
	p24 complex: Tretramers (αβγδ) or Heterodimers (2β2δ) α-members: Erp1, Erp5 and Erp6 β-member: Emp24 γ-members: Erp2, Erp3 and Erp4 δ-member: Erv25	p24 complex: Tretramers (αβγδ) or Heterodimers (2β2δ) [24] α-members: p24α1, p24α2 and p24α3 β-member: p24β1 γ-members: p24γ1, p24γ2, p24γ3, p24γ4 and p24γ5 δ-member: p24δ1
**COPII vesicle formation**		
COPII coat inner layer	Lst1/Sec23	Sec24C-D/Sec23
COPII coat outer layer	Sec13/Sec31	Sec13/Sec31

## Abbreviations

GPI	Glycosylphosphatidylinositol
GPI-AP	Glycosylphosphatidylinositol-anchored protein
ER	Endoplasmic reticulum
ERES	Endoplasmic reticulum exit site
EtNP	Ethanolamine phosphate
Man	Mannose
GlcN	Glucosamine
